# Reconsidering Causal Interpretation of Stair-Climbing Epidemiology and Necessary Precautions for Cardiovascular Patients

**DOI:** 10.3390/healthcare14111531

**Published:** 2026-06-01

**Authors:** Domenico Di Baggio, Vincenzo Romano Spica

**Affiliations:** Department of Movement, Human and Health Sciences, University of Rome “Foro Italico”, 00135 Rome, Italy

**Keywords:** stair climbing, cardiovascular risk, physical activity, anaerobic exercise, epidemiology, public health, lifestyles, health education

## Abstract

**Highlights:**

**What are the main findings?**

- Evidence suggests that regular stair climbing could be a potential marker or mediator of cardiovascular and metabolic health.
- Interpretation of the results may hide a pattern consistent with reverse causation.

**What are the implications of the main findings?**

- Stair climbing engages both neuromotor and cardiovascular systems, involving also anaerobic components at higher intensities that may pose a risk for susceptible individuals.
- Additional studies are needed to validate the mechanisms and predictive value of stair climbing as a preventive habit in different subjects, including the elderly.
- The application of stair climbing for preventive purposes may pose greater risks than benefits if not properly individualized within an Adapted Physical Activity framework.

**Abstract:**

**Background:** Stair climbing is increasingly associated with reduced cardiovascular risk, including a lower incidence of atrial fibrillation and mortality. **Objectives:** In this opinion paper, we aim to critically evaluate whether these associations reflect a true protective effect or, rather, underlying differences in baseline health status. **Methods:** After performing a narrative synthesis of recent observational studies and meta-analyses, we prepared this thought-provoking “Opinion paper” considering current clinical guidelines on cardiovascular risk and exertional stress. **Results:** Habitual stair climbing is consistently associated with reduced risks of atrial fibrillation, cardiovascular mortality, and all-cause mortality, with apparent dose–response relationships. However, these findings may be influenced by reverse causation, as individuals with greater functional capacity are more likely to engage in stair climbing. **Conclusions:** additional research is required to elucidate the underlying mechanisms and define optimal conditions under which stair climbing may act as a causal protective factor, before its widespread promotion in public health campaigns.

## 1. Opinion

Accumulating research is suggesting that stair climbing may be associated with reduced cardiovascular risk, including a lower incidence of atrial fibrillation (AF) [1]. Based on over 451,000 UK Biobank participants, it has been reported that climbing 10–50, 60–100, 110–150, and ≥160 stairs per day is associated with progressively lower hazard ratios for AF when compared with not climbing stairs, with stronger effects among individuals with lower cardiorespiratory fitness. These findings align with previous analyses demonstrating reduced cardiovascular and all-cause mortality among habitual stair climbers, including a 24% reduction in all-cause mortality and a 39% reduction in cardiovascular mortality according to a 2024 ESC meta-analysis involving 480,479 participants. A systematic review published in 2024 further reported significant reductions in cardiovascular (RR: 0.65) and all-cause mortality (RR: 0.70) associated with stair climbing [2,3,4].

However, despite the consistency of these observational findings, causality remains uncertain. Stair climbing is a demanding, high-intensity, often anaerobic activity. Thus, individuals who routinely climb stairs may simply represent a population with higher baseline functional capacity, fewer symptoms, and a lower subclinical disease burden. Conversely, those who avoid or discontinue stair climbing may already be experiencing early physiological decline, even if it has not clinically manifested. In the UK Biobank prospective cohort, for example, individuals who stopped climbing stairs exhibited a 32% increased risk of ASCVD compared with non-climbers—a pattern consistent with reverse causation, whereby reduced capacity for exertion may signal preclinical deterioration rather than mediate cardiovascular protection. Dose–response gradients may similarly reflect an unmeasured continuum of vitality or frailty rather than an exposure/effect relationship, a limitation intrinsic to self-reported physical activity measures.

It is therefore essential to contextualize the potential benefits of stair climbing with the possible risks for individuals at elevated cardiovascular risk, particularly because stair climbing induces abrupt increases in myocardial oxygen demand, blood pressure, and heart rate. High-intensity exertion may provoke ischemia or arrhythmia in patients with coronary artery disease, as outlined in the 2025 ACC/AHA considerations on cardiovascular abnormalities and exertional stress. Likewise, individuals with valvular heart disease—especially aortic stenosis—may be at risk of syncope, arrhythmias, or hemodynamic instability during strenuous or anaerobic efforts, as highlighted in the 2025 ESC/EACTS Guidelines for Valvular Heart Disease [5,6,7,8,9]. Recent work has shown that patients with coronary artery disease require supervision during high-intensity stair-climbing protocols, reflecting the physiological stress imposed by this activity. For these reasons, recommendations regarding stair climbing should include appropriate medical caveats for individuals with known or suspected cardiovascular disease.

## 2. Stair-Climbing Advantages: Movement Characteristics and Health Benefits of Habitual Stair Climbing

Stair climbing represents a readily accessible form of physical activity that integrates both aerobic and anaerobic components, making it a unique modality within daily-life exercise. From a physiological perspective, stair ascent is predominantly an aerobic activity when performed at a moderate pace, as it relies on oxidative metabolism to sustain repeated muscular contractions over time. The benefits are well established and described in different studies, so stair climbing can be considered an effective and easily accessible approach to promoting a kind of adapted physical activity in the population [2,3,4].

From a physiological perspective, it can be considered a high-intensity activity, as it may exceed 6 METs and 60% of VO_2_max, thereby eliciting the well-known metabolic benefits associated with exercise performed under similar conditions, provided it is practiced regularly and in a balanced manner appropriate for the individual’s physical condition and training level [10,11,12,13]. In other words, stair climbing should not be introduced abruptly as a major change to daily habits but rather adopted gradually through a progressive approach tailored to an individual’s personal and clinical profile, including age, sex, fitness level, prior sports experience, and any potential subclinical conditions that may pose risks, ranging from cardiovascular to neurological or orthopedic conditions. Additionally, an important factor to consider is an individual’s attitude toward and personal enjoyment of this type of physical activity, constituting a key element in inducing and sustaining the benefits of a lifestyle that includes stair climbing as part of one’s daily physical activity for health promotion.

As a lifestyle behavior, habitual stair climbing is categorized as incidental physical activity, embedded within daily routines rather than structured exercise programs. This aspect enhances adherence and sustainability, especially in sedentary populations, and aligns with current public health recommendations emphasizing the accumulation of moderate-to-vigorous physical activity throughout the day [14].

From a cardiovascular standpoint, stair climbing induces acute increases in heart rate, stroke volume, and systolic blood pressure, thereby improving cardiorespiratory fitness (CRF) over time. Enhanced CRF is strongly associated with a reduced risk of cardiovascular disease and mortality [15]. Regular stair use has also been linked to improved endothelial function and reduced arterial stiffness, contributing to better vascular health [16].

In terms of musculoskeletal benefits, stair climbing requires repeated concentric and eccentric contractions of major lower-limb muscle groups, including the quadriceps, gluteal muscles, and calf muscles. This increases muscle strength and tone, particularly in the lower body, and may help counteract sarcopenia in aging populations [17]. Additionally, the dynamic and weight-bearing nature of the activity helps maintain bone mineral density, reducing the risk of osteoporosis.

Stair climbing also helps to improve balance and neuromotor coordination, as it involves continuous postural adjustments and proprioceptive feedback. These adaptations are particularly relevant for fall prevention in older adults [18].

Metabolically, habitual stair climbing enhances glucose uptake and insulin sensitivity, partly due to increased skeletal muscle activity and mitochondrial function. Studies have shown improvements in lipid profiles, including reductions in LDL cholesterol and increases in HDL cholesterol, as well as favorable effects on body composition [19].

Finally, stair climbing represents a key tool in health promotion interventions based on an adapted physical activity approach in several frameworks. Within this context, it is important to distinguish between vigorous stair-climbing protocols, which are typically structured and performed at higher intensities; habitual daily stair use, which reflects spontaneous, low-threshold physical activity integrated into everyday life; and supervised exercise interventions in clinical populations, which are individually prescribed and monitored to ensure safety, appropriateness, and therapeutic efficacy.

Despite the evidence-based benefits of stair climbing, it is important to recognize that individuals who regularly climb stairs may already have higher baseline fitness levels, potentially confounding observed associations in clinical trials and epidemiological studies. Nonetheless, the integration of stair climbing into daily routines represents a practical and time-efficient strategy for improving multiple domains of health.

## 3. Stair-Climbing Risks: Cardiovascular, Musculoskeletal, and Neurological Threats of Stair Climbing

Despite its recognized benefits, stair climbing is a high-intensity activity that may pose clinically relevant risks, particularly in individuals with underlying or undiagnosed conditions. Therefore, it should be prescribed and introduced with caution, with the approach tailored and individualized on a case-by-case basis. Moreover, health education campaigns aimed at promoting stair climbing across diverse populations should be implemented with caution, ensuring that all necessary information is provided to support safe behavioral change, including guidance for those rare individuals who may unknowingly be at risk. In such cases, introducing an unfamiliar or inadequately tailored physical effort could trigger health issues, particularly when it exceeds an individual’s habitual level of physical conditioning.

Indeed, the evidence-based benefits must be balanced by the control and prevention of potential adverse effects. Due to the relatively high intensity per unit of time, often exceeding 8–10 metabolic equivalents (METs), stair climbing also engages anaerobic pathways, particularly during rapid or prolonged climbs, leading to lactate accumulation and increased cardiovascular strain [10,11]. Reaching the anaerobic threshold at this intensity varies greatly and depends on an individual’s baseline cardiorespiratory fitness, particularly their VO_2_ max. Moreover, for elderly individuals or those with cardiovascular conditions, the absolute intensity required to trigger anaerobic metabolism and cardiovascular strain can be significantly lower. Stair climbing qualifies as high-intensity physical activity, as it can exceed ≥6 METs (or ≥60–70% VO_2_max) and elicit high cardiorespiratory strain. Its use in fitness testing further supports the notion that it exerts a substantial physiological demand, consistent with high-intensity classifications [12,13].

From a cardiovascular standpoint, it has long been well documented that stair climbing induces rapid hemodynamic changes, including marked increases in heart rate, systolic blood pressure, and myocardial oxygen consumption [20]. As a form of vigorous intermittent physical activity, it may acutely elevate afterload and cardiac workload, especially in individuals with hypertension or reduced vascular compliance. In susceptible populations, such abrupt exertion can trigger myocardial ischemia, particularly in those with occult coronary artery disease, due to a mismatch between oxygen supply and demand.

Moreover, high-intensity exertion has been associated with a transient increase in the risk of acute cardiovascular events, including arrhythmia and sudden cardiac events, especially in untrained individuals or those with pre-existing cardiac abnormalities. Contemporary exercise guidelines emphasize that vigorous activity can precipitate atrial and ventricular arrhythmias in predisposed individuals, particularly when baseline screening is lacking [21]. Importantly, individuals who are unable or unwilling to climb stairs may already have reduced functional capacity or underlying disease, suggesting a potential overlap between risk exposure and reverse causation.

Recent studies highlight that the acute physiological demands of stair ascent can expose latent vulnerabilities across not only cardiovascular but also musculoskeletal or neurological systems [22,23,24].

The musculoskeletal risks are also significant. Stair climbing involves repetitive high-load joint flexion and extension, particularly at the knee and ankle, increasing stress on articular cartilage and periarticular structures. This may exacerbate osteoarthritis symptoms or contribute to overuse injuries, especially in overweight or older individuals. Additionally, stair negotiation is a well-documented context for accidental injuries, including sprains, ligament tears, and joint instability episodes. Falls on stairs represent a major public health concern. Compared to level-ground falls, stair-related falls are associated with a higher incidence of severe trauma, including fractures and head injuries. In individuals with osteoporosis, even low-energy trauma can result in fragility fractures, particularly of the hip or vertebrae, with substantial morbidity and mortality implications [25].

Neurologically, stair climbing requires intact balance, coordination, and executive motor control. Subclinical impairments—such as early neurodegenerative disorders, peripheral neuropathies, or vestibular dysfunction—can compromise gait stability and increase fall risk. Emerging evidence suggests that even mild deficits in gait variability and postural control may precede overt neurological disease and significantly elevate the risk of missteps and falls, particularly in complex tasks like stair descent.

Additionally, exertion-related events such as orthostatic hypotension, transient arrhythmias, or cerebrovascular insufficiency may manifest during stair climbing, leading to dizziness or syncope and further increasing injury risk. These risks are particularly relevant in regard to older adults or individuals with poorly controlled hypertension.

In conclusion, while stair climbing is an effective and accessible form of physical activity, it also represents a physiologically demanding task that may unmask or exacerbate underlying conditions. Careful risk stratification and the formation of individualized recommendations remain essential before it can be promoted universally. Moreover, as a purely speculative and hypothetical consideration, it may also be suggested that individuals who prefer not to climb stairs might not do so due to perceived difficulties or mild underlying issues such as dizziness, balance insecurity, an inadequate vascular response to an unaccustomed level of physical exertion, or simple knee joint problems. These factors may, often unconsciously, discourage individuals from climbing stairs and limit access to the activity’s potential benefits, not because of laziness or a lack of awareness of its advantages but rather due to bodily signals suggesting that a different type of physical activity may be more appropriate for their specific condition.

## 4. Conclusions

In conclusion, recent and rigorous epidemiological analyses are adding valuable evidence to an increasing body of research on how stair climbing may serve as a potential marker or mediator of cardiovascular health. Nevertheless, observational data cannot fully distinguish between causal protection and the influence of underlying functional reserves. Public health messages should therefore avoid implying universal benefits and should explicitly caution that stair climbing may pose non-negligible risks for individuals with coronary, valvular, or arrhythmogenic conditions, including those with clinically overt or subclinical cardiovascular disease, as well as other potentially relevant conditions such as subclinical neurological or osteotendinous disorders.

Finally, the widespread dissemination of the recommendation to use stair climbing as a health-promoting strategy could be misinterpreted or applied inappropriately by the general population. Appropriate communication is a key point. To simplify and illustrate examples commonly observed in everyday health promotion practices related to physical activity interventions, it is worth noting that individuals may occasionally misinterpret recommendations, potentially leading to inappropriate behavioral responses. For instance, some sedentary individuals might overexert themselves by carrying heavy shopping bags while deliberately avoiding elevators in daily life or by rushing up stairs at a fast pace, thereby imposing an uneven and potentially excessive load on both the musculoskeletal and cardiovascular systems. Similarly, older adults with subclinical, asymptomatic cardiovascular, neurological, or musculoskeletal conditions may modify their lifestyles and daily habits by engaging intensively in stair climbing, under the assumption that it confers significant cardiovascular or metabolic benefits. Moreover, even individuals who are unaware of the fact that they have conditions such as hypertension, or who are susceptible to hypertensive crises, may experience acute adverse events because of this type of physical exertion. Additional at-risk situations include undiagnosed osteoporosis or balance disorders, which may increase the likelihood of microfractures, falls or injuries. Therefore, although stair climbing represents an evidence-based and effective tool for health promotion, and although the available epidemiological evidence and related recommendations may appear straightforward, they should be interpreted and communicated with caution. Recommendations should always be individualized, and the promotion of physical activity must be tailored to each person’s health status and functional capacity.

Further research employing objective measures of physical activity and interventional study designs is warranted to clarify causal relationships and generate robust, evidence-based recommendations. Such evidence will also support the development of appropriate educational strategies for both the general population and healthcare professionals involved in health promotion campaigns for individuals of different ages and with different health conditions.

## Data Availability

No new data were created or analyzed in this study. Data sharing is not applicable to this article.

## Abbreviations

The following abbreviations are used in this manuscript:

AF	Atrial Fibrillation
CRF	Cardiorespiratory Fitness
MET	Metabolic Equivalent of Task
